# The Relationship Between Pet Attachment and Pet Loss Grief in Chinese Undergraduates: A Conditional Process Model

**DOI:** 10.3390/bs15040431

**Published:** 2025-03-27

**Authors:** Yangting Wu, Jingjing Song

**Affiliations:** Department of Psychology, School of Education, China University of Geosciences (Wuhan), Wuhan 430074, China

**Keywords:** pet attachment, pet loss grief, deliberate rumination, continuing bonds, disenfranchised grief

## Abstract

Pet owners may experience grief following the loss of their pets, stemming from the disruption of the emotional bond between them and their pets. This study aimed to examine the relationship between pet attachment and pet loss grief. A total of 160 college students who had experienced pet loss completed measures assessing pet attachment, deliberate rumination, pet loss grief, continuing bonds, and disenfranchised grief before their mental health course in China. The results indicated that deliberate rumination mediated the relationship between pet attachment and pet loss grief. Additionally, continuing bonds and disenfranchised grief moderated the relationship between deliberate rumination and pet loss grief. Specifically, deliberate rumination was positively associated with pet loss grief when continuing bonds were low and disenfranchised grief was high (β = 0.33, *p* < 0.01), while it was negatively associated with pet loss grief when continuing bonds were high and disenfranchised grief was low (β = −0.32, *p* < 0.01).

## 1. Introduction

Pet loss grief refers to a distressing emotional response triggered by the death of a pet, or by life events such as divorce, breakups, or changes in living arrangements that lead to the loss of pet companionship and the inability to maintain cohabitation with the pet. This grief stems from the disruption of the emotional bond between a person and their pet (Cowling et al., 2020). Approximately 30% of pet owners experience intense grief following pet loss. This grief can increase psychological distress and the risk of mortality (Lee, 2020; Stroebe et al., 2007). Therefore, it is necessary to explore the factors related to pet loss grief. Previous studies have demonstrated that strength of pet attachment is significantly associated with the intensity of pet loss grief (Field et al., 2009; Jordan & Vonk, 2024). We aimed to further investigate when and how the pet attachment influences pet loss grief, while also exploring factors that alleviate and reduce such emotional distress. The findings have the potential to help millions of pet owners effectively manage these profound psychological stressors.

### 1.1. Pet Attachment and Pet Loss Grief

The human–pet bond refers to a special emotional connection and mutual relationship between humans and pets. For many families, people perceive their pets as family members, friends, and children, rather than animals (Brooks et al., 2018; Kemp et al., 2016). Pets provide companionship, alleviate individual stress, and facilitate social interactions, promoting better physical health (e.g., lower blood pressure) and psychological resilience (Ennis & Majid, 2021; Yang et al., 2017). As emotional dependence increases, the human–pet bond develops into a deeper attachment relationship, resembling that observed in humans (Field et al., 2009; Leonhardt-Parr & Rumble, 2024). There are two different forms of attachment: attachment bond and attachment orientation. Attachment orientation refers to the relatively stable behavioral patterns and psychological tendencies that an individual develops in the relationship with their pet. This includes secure attachment, anxious attachment, and avoidant attachment (Mikulincer & Shaver, 2010). In contrast, pet attachment bond describes the strength of emotional bonds between pet owners and pets. A systematic review, which retrieved 40 articles in the human–pet attachment field, found that the majority of studies focused on attachment bond, whereas only seven (18%) of the studies focused on attachment orientations (Jordan & Vonk, 2024). The present study focuses on pet attachment strength because it is a quantifiable variable. Pet attachment strength can more effectively clarify how and when pet attachment relates to pet loss grief.

The strength of pet attachment may shape an individual’s emotional response to pet loss. A study examining individuals who had lost their pets within the past few years showed that the strength of pet attachment was positively correlated with pet loss grief (Cowling et al., 2020). Pet owners with stronger attachment to their pets tend to experience more intense grief following their loss (Amiot & Bastian, 2015; Field et al., 2009; Jordan & Vonk, 2024).

Cultural differences exist between Eastern and Western countries regarding pet perceptions. In Western countries, pets are typically regarded as important members of the family, while in Eastern countries, particularly in China, although pets are acknowledged as part of the family, there is a comparatively stronger tendency to perceive them as companions or even accessories (Chen et al., 2018). These cultural differences may lead to variations in pet attachment strength, the intensity of pet loss grief, and the extent to which pet attachment is associated with grief responses. However, in China, empirical research examining the relationship between pet attachment and pet loss grief remains limited. Therefore, this study focuses on Chinese pet owners and investigates the association between pet attachment and pet loss grief. We hypothesize that pet attachment is positively correlated with pet loss grief (H1).

### 1.2. Mediating Role of Deliberate Rumination

Previous studies have identified several factors that mediate the relationship between pet attachment and pet loss grief, including the anthropomorphization of pets (Behler et al., 2020), emotion regulation strategies (Kim, 2022), and separation distress (H. J. Park & Jeong, 2022). Cognitive processing theory suggests that the way individuals process information is associated with their emotional and behavioral responses (Resick et al., 2017). Accordingly, the perceptions and cognitive appraisals associated with pet loss are likely to be associated with the experience of pet loss grief. Rumination, conceptualized as the repetitive and passive focus on a distressing event and its consequences (Watkins, 2008), is a cognitive process through which individuals engage in recurrent reflection, evaluation, and reappraisal of relevant information. Therefore, rumination may serve as a mediating mechanism between the pet attachment and pet loss grief. However, to our knowledge, no prior research has directly examined the mediating role of rumination in this relationship.

Rumination comprises two types: intrusive and deliberate. Intrusive rumination refers to the process in which individuals repetitively and unconsciously think about and dwell on negative emotions, experiences, or problems (Nolen-Hoeksema & Davis, 2004). Individuals with strong intrusive rumination tend to focus on the negative aspects of pet loss while neglecting to address distressing emotions. As a result, they might experience prolonged grief (Yousefi & Ashouri, 2023). According to response styles theory, rumination is a maladaptive coping strategy that prompts individuals to repeatedly contemplate their loss, leading to maladjustment during bereavement (Nolen-Hoeksema, 2001, 2003). The rumination as avoidance hypothesis suggests that rumination is a form of cognitive avoidance strategy, and it interferes with the acceptance of the loss, prevents individuals from integrating memories of the loss with existing memories, and exacerbates grief (Stroebe et al., 2007; Eisma & Stroebe, 2017). Conversely, deliberate rumination refers to the intentional and goal-directed cognitive process through which individuals actively revisit and re-evaluate the event (Clout et al., 2024). It has been demonstrated that deliberate rumination can help individuals gain a deeper understanding of their emotions, facilitate grief processing, enhance psychological resilience, improve self-awareness, enhance problem-solving abilities, lead to posttraumatic growth, and have a positive impact on their psychological and behavioral development (García et al., 2015; Zięba et al., 2022). Previous research also found that deliberate rumination plays a crucial role in the recovery process of women who experienced pregnancy termination due to fetal abnormality (Ye et al., 2024).

A previous study suggests that pet loss, as a significant stressful event, may elevate both deliberate and intrusive rumination (Huh et al., 2020). The higher the level of pet attachment, the greater the emotional trauma of losing a pet, which is accompanied by increased separation anxiety in pet owners (H. J. Park & Jeong, 2022). The negative emotional experience may lead to heightened rumination. Pet owners often recall and miss their pets and try to understand, process, and adapt to the emotional shock of loss (Albuquerque et al., 2017). Based on the literature reviewed (Nolen-Hoeksema, 2001, 2003; Clout et al., 2024; Zięba et al., 2022), we focus on the deliberate rumination and analyze the mediation role of deliberate rumination in the relationship between pet attachment and pet loss grief. We hypothesize that pet attachment is positively associated with deliberate rumination during pet loss (H2a), and that deliberate rumination is negatively associated with pet loss grief (H2b).

### 1.3. Conditions Under Which Pet Attachment Associates with Pet Loss Grief

Previous studies have examined the moderating effect of self-compassion and separation distress in the relationship between pet attachment and pet loss grief (Bussolari et al., 2021; H. J. Park & Jeong, 2022). It is necessary to explore other potential moderating factors between pet attachment and pet loss grief.

Continuing bonds refer to behaviors that maintain an emotional connection with the departed (Habarth et al., 2017). It includes activities such as conducting funeral ceremonies, preserving mementos, and engaging in reminiscence (Habarth et al., 2017). Field and Filanosky (2009) divided continuing bonds into internalized and externalized dimensions. Internalized continuing bonds primarily involve contemplation, reflection, and reminiscence of the deceased pet, which are similar to deliberate rumination. Specifically, while deliberate rumination focuses on the cognitive processing of negative emotions, internalized continuing bonds emphasize the ongoing relationship with the deceased pet.

Continuing bonds may moderate the relationship between pet attachment and pet loss grief. There are contrary results related to the role of continuing bonds in pet loss grief management. Some researchers showed that continuing bonds facilitate the grief coping, and have a positive impact on individuals’ health and well-being (Habarth et al., 2017; Jones et al., 2023; Wolf et al., 2024). Packman et al. (2011) surveyed individuals who recently experienced pet loss, finding that greater reliance on continuing bonds coping strategies correlated with lower levels of grief and mental health symptoms. Furthermore, the lack of standardized memorialization procedures may cause pet owners to experience higher levels of grief (Cleary et al., 2022). However, some researchers have suggested that continuing bonds may exacerbate grief and increase somatization symptoms (Lavorgna & Hutton, 2019; Schmidt et al., 2020; Tzivian et al., 2014). We hold the view that continuing bonds afford pet owners the opportunities to yearn for and reminisce about their departed pets. This may intensify grief in the short term. Nevertheless, over time, these bonds may facilitate a healthier grieving process by enabling them to bid a proper farewell to their beloved pets (Hughes & Lewis Harkin, 2022). Based on the reviews above (Hughes & Lewis Harkin, 2022; Schmidt et al., 2020), we hypothesize that continuing bonds moderate the relationships between pet attachment and pet loss grief (H3a). Specifically, the positive association between pet attachment and pet loss grief is expected to be weaker when continuing bonds are strong compared to when they are weak.

Continuing bonds may also moderate the relationship between deliberate rumination and pet loss grief. Some pet owners may feel guilty that they did not provide sufficient companionship to their pets during their lifetime or fail to detect the illness of their pets earlier and did not offer the best medical care (Zisook & Shear, 2009). Under conditions of strong continuing bonds, the pet owners maintain an emotional connection with their lost pets. The continuing bonds offer an opportunity to address unresolved relational conflicts, alleviate feelings of guilt associated with pet loss (Suhail et al., 2011). In this context, deliberate rumination primarily involves missing the pet and expressing gratitude for its companionship. It can facilitate acceptance of the pet’s passing and alleviate the grief of pet loss. Conversely, when continuing bonds are weak, pet owners engage less in action to maintain a connection with their lost pet. In such cases, deliberate rumination may intensify feelings of guilt and regret, particularly if pet owners believe that they failed to provide sufficient companionship or medical care during the pet’s lifetime. This may lead to heightened pet loss grief. Therefore, we propose that continuing bonds moderate the relationship between deliberate rumination and pet loss grief (H3b). Specifically, for pet owners with strong continuing bonds, deliberate rumination is negatively associated with pet loss grief. In contrast, for pet owners with weak continuing bonds, deliberate rumination is positively associated with pet loss grief.

Disenfranchised grief refers to grief that society restricts or does not recognize as legitimate (APA, 2022). Friends and family of pet owners may consider pet loss grief unnecessary, leaving owners feeling unrecognized and unsupported (Kemp et al., 2016; Tzivian et al., 2014). Even when loved ones acknowledge the pet loss grief, pet owners may hesitate to express their grief and avoid seeking support because of fear of misunderstanding, contributing to self-imposed disenfranchised grief (Wong et al., 2017). Research suggests that one-third of pet owners have experienced disenfranchised grief (Brown et al., 2023).

Disenfranchised grief might moderate the relationship between pet attachment and pet loss grief. Previous studies have shown that disenfranchised grief limits pet owners’ ability to express their pet loss grief (Cordaro, 2012; Spain et al., 2019), thereby exacerbating social isolation, hindering grief resolution (Adrian & Stitt, 2017; Packman et al., 2014), and increasing levels of depression and anxiety (Adrian & Stitt, 2017; Bussolari et al., 2021). Social support theory suggests that social support can buffer the negative impact of life stressors on individuals’ physical and mental health (Cohen & Wills, 1985). When pet owners are in a supportive environment, they experience lower levels of guilt and depression (Dunn et al., 2005) and are less likely to develop complicated grief (Packman et al., 2014; Tzivian et al., 2014). Furthermore, previous research also indicates that perceived social support plays a moderating role in the relationship between pet anthropomorphization and pet loss grief (Behler et al., 2020). Thus, we hypothesize that disenfranchised grief moderates the relationship between pet attachment and pet loss grief (H4a), and that the association between pet attachment and pet loss grief is weaker when disenfranchised grief is low than when it is high.

Moreover, disenfranchised grief may moderate the relationship between deliberate rumination and pet loss grief. When disenfranchised grief is high, a pet owner’s deliberate rumination and longing for their pets may not be acknowledged or understood by others. Deliberate rumination may intensify feelings of social isolation and loneliness, reinforcing thoughts such as “Why do others not understand me?” and ultimately exacerbating grief (Ciesla & Roberts, 2007). In contrast, when disenfranchised grief is low, pet owners are more likely to receive social support from family and friends. In this supportive context, deliberate rumination could help individuals better understand the experience of pet loss, facilitate grief management and coping, explore the meaning of life in the event of loss, and cultivate appreciation for the present. Thus, we hypothesize that deliberate rumination is positively associated with pet loss grief when disenfranchised grief is high, whereas it is negatively associated with pet loss grief when disenfranchised grief is low (H4b).

### 1.4. Conceptual Framework

**Figure 1:**
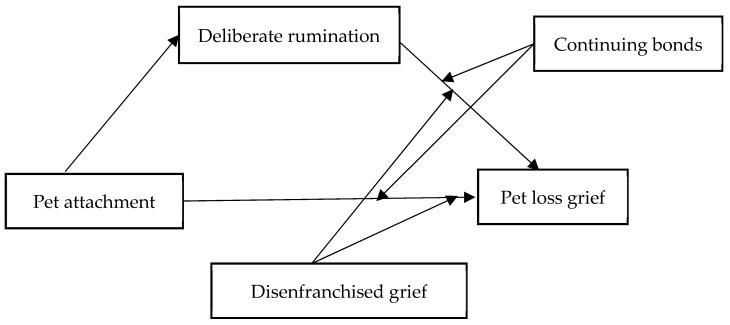
The relationship between pet attachment and pet loss grief: The mediating role of deliberate rumination, and the moderating role of continuing bonds and disenfranchised grief.

According to the reviews above (Brown et al., 2023; Ennis & Majid, 2021; Wolf et al., 2024; Zięba et al., 2022), previous research has the following gaps of knowledge. Firstly, research on pet loss grief has predominantly focused on western countries, with limited research in China, which is primarily concentrated in Hong Kong (Wong et al., 2017). Given China’s rapidly expanding pet market, further research in this context is warranted. Secondly, previous research examining the relationship between rumination and pet loss grief did not distinguish between deliberate rumination and intrusive rumination, limiting a nuanced understanding of their differential effects. Thirdly, previous studies analyzed the linear relationship between pet attachments and pet loss grief, but fewer studies further explored the conditions under which pet attachment associated with pet loss grief. It is necessary to develop a theoretical model to provide a more comprehensive understanding of the complex relationship between pet attachment and pet loss grief. In summary, we intend to analyze the relationship between pet attachment and pet loss grief in a Chinese sample and to further analyze the mediating role of deliberate rumination, as well as the moderation role of “disenfranchised grief” and “continuing bonds” (refer to Figure 1).

## 2. Materials and Methods

### 2.1. Participants

Participants were opportunistically recruited from a university in central China. We selected three classes of college mental health psychology, which were attended by students from different faculties across the university. Before the commencement of these classes, an experimenter introduced the research objectives, and the participants voluntarily scanned a QR code to complete an online survey. A total of 460 university undergraduates participated in the investigation. After excluding the participants who had not experienced pet loss and those with incomplete responses, a final sample of 160 valid participants was obtained (men = 97, women = 63). The age of participants ranged from 18 to 24 (M = 18.18, SD = 1.23). Most participants (n = 126, 78.8%) were single (i.e., unmarried or not in a romantic relationship). The duration of pet ownership ranged from 1 month to 14 years (M = 3.34, SD = 2.94). The time since pet loss ranged from 1 to 12 years (M = 6.02, SD = 8.41). Among the participants, the three most frequently reported pets that had been lost were dogs (n = 104, 65%), cats (n = 26, 16%), and turtles (n = 10, 6%). Regarding the reasons for pet loss, 58 pets were lost, 27 pets died due to illness or other causes, 28 died of old age, and 26 experienced traumatic events (e.g., traffic accidents).

### 2.2. Procedures

Ethical approval for this study was obtained from the ethics committee of the first author’s institution (IRB protocol number: cug-ecdp-24-09-02). We recruited participants through university mental health courses. Before data collection, participants firstly filled out an informed consent form, then completed demographic information and relevant questionnaires. To ensure standardized data collection, a trained researcher read aloud a guidebook outlining the study’s purpose, participant requirements (e.g., answering questions carefully), and participant rights (e.g., anonymity of responses and the ability to withdraw from the study at any time without penalty). Participants took approximately 10 min to complete all questionnaires and received partial credit. The estimated minimum sample size required to detect a medium effect was 78 (*p* = 0.05).

### 2.3. Research Tools

#### 2.3.1. Pet Attachment Questionnaire (PAQ)

The Pet Attachment Questionnaire (PAQ) developed by Albert and Bulcroft (1988) has been widely used to measure the degree of pet attachment. This scale has been widely used to measure pet attachment and has demonstrated good reliability and validity (Ciacchella et al., 2025; Zilcha-Mano et al., 2011). It consists of 9 items, for example: “I feel closer to my pet than many of my friends”. A five-point scale was used, with 1 = totally disagree, 5 = totally agree. The higher score indicated the high level of pet attachment. The Cronbach’s alpha was 0.87 in this study.

#### 2.3.2. The Pet Bereavement Questionnaire (PBQ)

The PBQ (Hunt & Padilla, 2006) was used to assess the degree of pet loss grief. It consists of 16 items, encompassing three dimensions of sadness (I am very upset about my pet’s death), anger (I feel angry that I can’t save my pet), and guilt (I should notice that something bad have happened to my pet). This scale is widely adopted for measuring pet loss grief, exhibiting good reliability and validity (Testoni et al., 2017). A four-point scale was used, with 0 = totally disagree, 3 = totally agree. Higher scores reflected greater pet loss grief. The scale demonstrated good reliability, with a Cronbach’s alpha of 0.90 in this study.

#### 2.3.3. Chinese Version of Event-Related Rumination Questionnaire

The Chinese version of the Event-Related Rumination Questionnaire (C. Dong et al., 2013) was used to measure deliberate rumination. This scale has been widely utilized to assess deliberate rumination and has exhibited good reliability and validity (C. Wang et al., 2022). We modified some descriptions to make it suitable for measuring the deliberate rumination of pet loss. It includes ten items, for example, I pondered if I could find something meaningful in the event of pet loss. Participants rated their rumination frequency within two weeks of losing a pet on a four-point Likert scale, with 0 = never, 3 = always. Higher scores suggested higher frequency of deliberate rumination. The scale showed good reliability, with a Cronbach’s alpha of 0.95 in this study.

#### 2.3.4. Continuing Bonds Interview Questionnaire (CBI)

The CBI (Field & Filanosky, 2009) is a semi-structured interview to assess the continuing bonds expressions and other experiences related to the loss of child. We modified this scale to assess CB expressions related to the death of a pet. It comprised 12 items, for example, belongings/possessions used to feel closer to the deceased pet. A four-point scale was used, with 1 = never, 4 = almost every day. The sum of each item was the total score, with higher scores indicated higher level of continuing bonds. It showed good reliability, with a Cronbach’s alpha of 0.90 in this study.

#### 2.3.5. Grief Experience Questionnaire (GEQ)

The “loss of support” dimension of the Grief Experience Questionnaire (Barrett & Scott, 1989) was used to measure disenfranchised grief. This scale is widely adopted for measuring disenfranchised grief, exhibiting good reliability and validity (Spain et al., 2019). It contains five items, for example, I think others didn’t want me to talk about the death. Responses were scored on a five-point scale, 1 = never, 5 = almost always, with higher score indicating greater extent of disenfranchised grief. The scale showed good reliability, with a Cronbach’s alpha of 0.94 in this study.

### 2.4. Data Analysis

Data were analyzed using SPSS 26.0 (Hayes, 2018). Descriptive statistics (means and standard deviations), correlation analysis (Pearson’s correlation coefficient), and the single-factor Harman test were conducted. To examine the conditional process model of pet attachment and pet loss grief (see Figure 1), we used the PROCESS macro in SPSS (Model 17). The analysis consisted of two models: The first model assessed the effect of pet attachment (independent variable) on deliberate rumination (mediator). The second model examined the effects of pet attachment, deliberate rumination, and the moderators (continuing bonds and disenfranchised grief) on pet loss grief (dependent variable). The significance of regression coefficients was tested using the Bootstrap method (Hayes & Scharkow, 2013).

## 3. Results

### 3.1. Descriptive Statistics and Correlation Analysis

The correlations among all variables were calculated using Pearson’s correlation coefficient (see Table 1). The results indicated that pet attachment was positively associated with pet loss grief (β = 0.54, *p* < 0.001), supporting H1. Pet attachment was also positively associated with deliberate rumination (β = 0.29, *p* < 0.001) and continuing bonds (β = 0.42, *p* < 0.001). Deliberate rumination, disenfranchised grief, and continuing bonds were significantly positively correlated with pet loss grief (β = 0.40, *p* < 0.001; β = 0.37, *p* < 0.001; β = 0.59, *p* < 0.001). Deliberate rumination was positively associated with disenfranchised grief (β = 0.29, *p* < 0.001) and continuing bonds (β = 0.50, *p* < 0.001). Disenfranchised grief was positively associated with continuing bonds (β = 0.23, *p* < 0.001).

The single-factor Harman test was used to assess the common method variance (Podsakoff et al., 2003). The results of exploratory factor analysis showed that the first factor explained 29.29% of the variance, which was less than the critical threshold of 40%. Thus, the data in this study did not exhibit significant common method bias.

### 3.2. The Conditional Processing Model Analysis

We used the PROCESS (Hayes, 2018) macro with model 17 in SPSS to examine the direct effects of pet attachment on pet loss grief, the mediating role of deliberate rumination, and the moderating role of disenfranchised grief and continuing bonds. Gender, age, and duration of pet loss were included as control variables.

Firstly, in the model assessing the relationship between the independent variable and the mediator, after controlling for demographic variables, pet attachment was significantly associated with deliberate rumination (β = 0.42, *p* < 0.001). H2a was supported. Additionally, gender was significantly associated with deliberate rumination, whereas age and duration of pet loss were not.

Secondly, in the model examining the independent variable, mediator, and moderators in relation to the dependent variable, the results indicated that deliberate rumination was not significantly associated with pet loss grief (β = 0.35, *p* > 0.05). Similarly, the relationship between disenfranchised grief and pet loss grief was also not significant (β = 0.26, *p* > 0.05). In contrast, pet attachment, continuing bonds were significantly and positively associated with pet loss grief (β = 0.82, *p* < 0.01; β =1.34, *p* < 0.01).

The interaction term of pet attachment and continuing bonds as well as the interaction term of pet attachment and disenfranchised grief were not significantly associated with pet loss grief (β = −0.01, *p* > 0.05; β = −0.02, *p* > 0.05), indicating that H3a and H4a were not supported. The interaction term of deliberate rumination and continuing bonds as well as the interaction term of deliberate rumination and disenfranchised grief were significantly negatively associated with pet loss of grief (β = −0.03, *p* < 0.01, β = −0.03, *p* < 0.05), supporting H3b and H4b (see Table 2). These results indicated that continuing bonds and disenfranchised grief moderated the relationship between deliberate rumination and pet loss grief but did not moderate the direct path of pet attachment and pet loss grief.

Since H3b and H4b were proved significant, we conducted a further analysis of the moderation effects. The results indicated that deliberate rumination was significantly positively associated with pet loss grief when continuing bonds were low and disenfranchised grief was high (β = 0.33, *p* < 0.01). Conversely, deliberate rumination was significantly negatively associated with pet loss grief (β = −0.32, *p* < 0.01) when continuing bonds were high and disenfranchised grief was low. These findings support H2b, suggesting that the mediating role of deliberate rumination has been partially confirmed. In summary, pet attachment was directly associated with pet loss grief and was also indirectly associated with pet loss grief through the mediating role of deliberate rumination, moderated by disenfranchised grief and continuing bonds.

## 4. Discussion

This study explored how and when pet attachment was associated with pet loss grief and analyzed the mediating role of deliberate rumination and the moderating roles of disenfranchised grief and continuing bonds. Consistent with previous research, we found that pet attachment was positively associated with pet loss grief (Field et al., 2009; Jordan & Vonk, 2024), and deliberate rumination mediated this relationship (An & Kim, 2022). Additionally, we further found that continuing bonds and disenfranchised grief acted as moderators in the relationship between deliberate rumination and pet loss grief, which have not been directly verified in previous research. Specifically, deliberate rumination was positively associated with pet loss grief when continuing bonds were low and disenfranchised grief was high, while it was negatively associated with pet loss grief when continuing bonds were high and disenfranchised grief was low.

This study presents the following innovations: (1) Research on pet loss grief has predominantly focused on Western countries (Wong et al., 2017; Fu et al., 2025). Our study extends this research by examining Chinese pet owners and analyzing their experiences of pet loss grief. (2) Prior studies related to the relationship between rumination and pet loss grief reported conflicting findings (Black et al., 2022; Lykins et al., 2024). It might be because they did not distinguish deliberate rumination from intrusive rumination. This current study specifically focused on deliberate rumination and examined its mediating role. (3) Previous research has primarily explored individual factors influencing pet loss grief (R. M. Park et al., 2023) and analyzed the linear relationship between pet attachments, rumination, continuing bonds, disenfranchised grief, and pet loss grief separately (Jessica et al., 2022; Spain et al., 2019). In contrast, we integrated these variables into a comprehensive theoretical model to explore when and how pet attachment is associated with pet loss grief. Our findings demonstrate that pet attachment was associated with pet loss grief through the mediating role of deliberate rumination, with continuing bonds and disenfranchised grief moderating the relationship between deliberate rumination and pet loss grief.

Consistent with previous research (Field et al., 2009; Jordan & Vonk, 2024; R. M. Park et al., 2023; Testoni et al., 2017), our research found a significant positive association between pet attachment and pet loss grief. Individuals with strong pet attachment are more likely to experience intense pet loss grief. Highly attached pet owners often spend substantial time with their pets, sometimes even more than with their family or friends. Their pets offer them unconditional emotional support and companionship, serving as a crucial source of social support for pet owners. Thus, the loss of a pet implies the deprivation of an important source of spiritual sustenance and companionship (Cowling et al., 2020).

Consistent with previous research demonstrating the mediating role of rumination in the relationship between pet attachment and post-traumatic growth (An & Kim, 2022; Rajkumar et al., 2024; Saltzman et al., 2024), our study found that pet attachment was indirectly associated with pet loss grief through deliberate rumination. Pet attachment was positively associated with deliberate rumination. For the pet owners with a high level of pet attachment, losing a pet, similar to losing an important family member, is a tremendously traumatic event. Such a traumatic event might disrupt psychological balance and cause confusion, helplessness, and a sense of loss of control. As a result, pet owners frequently reminisce about their pets, engaging in repeated deliberate rumination about past experiences (Albuquerque et al., 2017). Thus, the stronger the pet attachment, the more frequent the deliberate rumination about the deceased pet.

The current study found that continuing bonds and disenfranchised grief moderated the relationship between deliberate rumination and pet loss grief. Deliberate rumination was positively associated with pet loss grief when the continuing bonds were low and disenfranchised grief was high. When pet owners experience a lack of social recognition and support for their grief, and when they have limited opportunities to commemorate their deceased pet, high levels of deliberate rumination may exacerbate pet loss grief. Social support theory states that social support can help one cope with stress and maintain mental health (Zhang et al., 2025). However, societal perceptions that animals are inferior to humans and unworthy of the same level of emotional investment contribute to the lack of acknowledgment of pet loss grief (Meehan et al., 2017), thereby intensifying pet owners’ psychological distress. Moreover, prior research has emphasized that the goal of continuing bonds is not to end the relationship with the deceased, but to build a new and important connection with the deceased (Black et al., 2022; Field & Filanosky, 2009). The existence of continuing bonds such as funeral ceremonies and mementos can provide a way for individuals to communicate with and to bid farewell to their deceased pets. The continuing bonds help the pet owners gradually accept the departure of their loved ones, facilitating coping with grief. Thereby, continuing bonds enable pet owners to gradually confront the loss (G. Dong & Li, 2016).

Our results showed a negative correlation between deliberate rumination and pet loss grief when the continuing bonds were high and the disenfranchised grief was low. In contexts where pet owners’ grief is understood by others and where they can maintain bonds with their deceased pets, deliberate rumination can help pet owners cope with their grief. It is consistent with the findings of Papageorgiou and Wells (2001), who found that deliberate rumination can serve as a distress reduction strategy to facilitate grief coping under certain conditions. We speculate that, in a supported environment, deliberate rumination may focus on positive reflections and evaluations of the traumatic experience (Lindstrom et al., 2013). In such cases, deliberate rumination might help pet owners accept the reality of the pet’s death, consider potential positive aspects of the loss, and recognize the personal growth and benefits that emerge from this challenging and traumatic event (Folkman, 2008).

### 4.1. Implication

Our findings indicate that, despite varying cultural attitudes toward pet ownership, pet attachment remains positively correlated with pet loss grief among Chinese pet owners. Furthermore, the study highlights that disenfranchised grief and the absence of continuing bonds present significant challenges for Chinese pet owners, acting as primary drivers of their pet loss grief. To alleviate disenfranchised grief and promote continuing bonds, we propose the following actions: (1) Conduct lectures, workshops, and other activities on pet loss grief to raise awareness about the vital role pets play in human lives and the profound impact of pet loss on their owners. (2) Establish a pet loss grief support hotline to enhance the social support system. This service could provide psychological support and offer coping strategies for individuals grieving the loss of a pet. (3) Develop both online and offline mutual-aid communities where pet owners can share their experiences and feelings regarding pet loss, fostering a sense of solidarity and understanding. (4) The pet funeral industry should focus on standardization and humanization. Well-regulated pet funeral services can offer appropriate ways for pet owners to mourn their pets and provide them with comfort during a difficult time.

#### Limitations and Future Directions

This study has the following limitations: (1) Continuing bonds consist of various components: internalized and externalized (Habarth et al., 2017). Different forms of continuing bonds might have different effects on pet loss grief (Black et al., 2022). Future research needs to explore and compare the relationship between different continuing bonds and grief coping in pet loss. (2) There might exist cultural differences in the continuing bonds. In China, influenced deeply by religions such as Buddhism and Taoism, it is believed that death is the cycle or sublimation of life, emphasizing the rest of the soul and the well-being of the afterlife (Xu, 2025; S. Wang et al., 2024). People make paper money and paper ties (including various household items such as houses, cars, appliances, etc.) for the deceased, hoping that they can also live a comfortable life in another world. Thus, it is important for future studies to compare the continuing bonds in Eastern and Western cultures and compare their different associations with pet loss grief coping. (3) Deliberate and intrusive forms of rumination have different effects on pet loss grief (Huh et al., 2020; Okumura et al., 2024). Our study only analyzed the mediating role of deliberate rumination and did not compare the effect of deliberate rumination and intrusive rumination. (4) Different types of pets might have different roles, fulfilling different needs of pet owners and thus impacting the strength of pet attachment (Cowling et al., 2020). Future research could explore the moderating effect of pet type on the present research model. (5) Although we expected the moderators to influence the direct pathway between pet attachment and pet loss grief, the study results did not support this hypothesis. We propose that other key factors may better account for the conditions under which pet attachment is associated with pet loss grief. (6) The sample of this study is college students who were asked to recall pet loss events they had experienced in their past lives, but this event may have happened a long time ago. Future research can recruit pet owners who have just experienced pet loss to conduct targeted studies. (7) Our study is a cross-sectional design that investigates relevant variables through self-report surveys and cannot provide answers to causal relationships between variables.

## Figures and Tables

**Table 1:** Descriptive statistics and correlation analysis of all variables.

Variables	M	SD	1	2	3	4	5	6	7	8
1. Gender	1.42	0.49	-							
2. Age	18.18	1.23	0.10	-						
3. Duration of pet loss	6.02	2.91	0.08	−0.10	-					
4. Pet attachment	29.54	5.78	0.16 *	0.08	0.05	-				
5. Pet loss grief	40.94	8.09	0.13	0.13	0.00	0.54 ***	-			
6. Deliberate rumination	21.44	7.51	−0.18	0.03	−0.06	0.29 ***	0.40 ***	-		
7. Disenfranchised grief	9.23	4.41	−0.10	0.00	0.20 *	0.07	0.37 ***	0.29 ***	-	
8. Continuing bonds	23.59	6.45	−0.05	0.09	−0.15	0.42 ***	0.59 ***	0.50 ***	0.23 ***	-

Note: *** *p* < 0.001. * *p* < 0.05.

**Table 2:** The relationship between pet attachment and pet loss grief: The mediating role of deliberate rumination, and the moderating role of disenfranchised grief and continuing bonds.

Independent Variables	Predictor Variables	R	R^2^	F	B	t	*p*	LLCI	ULCI
Deliberate rumination	Pet attachment	0.37	0.14	6.10	0.42	4.26	0.00 ***	0.23	0.62
	Gender				−3.42	−2.96	0.01 **	−5.70	−1.13
	Age				0.14	0.46	0.76	−0.78	1.05
	Duration of pet loss				−0.13	−0.69	0.50	−0.52	0.25
Pet loss grief	Pet attachment	0.76	0.58	18.60	0.82	2.82	0.01 **	0.25	1.39
	Deliberate rumination				0.35	1.35	0.18	−0.17	0.87
	Disenfranchised grief				0.26	0.41	0.68	−1.00	1.52
	Pet attachment × Disenfranchised grief				−0.02	−0.95	0.34	−0.06	0.02
	Deliberate rumination × Disenfranchised grief				0.03	2.42	0.02 *	0.01	0.06
	Continuing bonds				1.34	3.42	0.00 ***	0.57	2.11
	Pet attachment × Continuing bonds				−0.01	−0.77	0.45	−0.03	0.01
	Deliberate rumination × Continuing bonds				−0.03	−2.55	0.01 **	−0.05	0.01
	Gender				1.73	1.86	0.07	−0.11	3.57
	Age				0.30	0.84	0.40	−0.41	1.01
	Duration of pet loss				−0.08	−0.49	0.62	−0.40	0.23

Note: *** *p* < 0.001. ** *p* < 0.01. * *p* < 0.05; R^2^ is used to explain the proportion of the variation in the dependent variable that is explained by the variation in the independent variable. LLCI represents the lower limit of the confidence interval, and ULCI represents the upper limit of the confidence interval. When the confidence interval includes 0, it indicates the correlation is not significant.

## Data Availability

The datasets generated and analyzed during the current study are not publicly available but are available from the corresponding author upon reasonable request.
